# Dynamic Rounds Chaotic Block Cipher Based on Keyword Abstract Extraction

**DOI:** 10.3390/e20090693

**Published:** 2018-09-11

**Authors:** Juan Wang, Qun Ding

**Affiliations:** 1 Electronic Engineering College, Heilongjiang University, Harbin 150001, China; 2 Electronic and Information Engineering College, Heilongjiang University of Science and Technology, Harbin 150027, China

**Keywords:** chaos, block cipher, dynamic encrypted rounds, abstract extraction

## Abstract

According to the keyword abstract extraction function in the Natural Language Processing and Information Retrieval Sharing Platform (NLPIR), the design method of a dynamic rounds chaotic block cipher is presented in this paper, which takes into account both the security and efficiency. The cipher combines chaotic theory with the Feistel structure block cipher, and uses the randomness of chaotic sequence and the nonlinearity of chaotic *S*-box to dynamically generate encrypted rounds, realizing more numbers of dynamic rounds encryption for the important information marked by NLPIR, while less numbers of dynamic rounds encryption for the non-important information that is not marked. Through linear and differential cryptographic analysis, ciphertext information entropy, “0–1” balance and National Institute of Science and Technology (NIST) tests and the comparison with other traditional and lightweight block ciphers, the results indicate that the dynamic variety of encrypted rounds can achieve different levels of encryption for different information, which can achieve the purpose of enhancing the anti-attack ability and reducing the number of encrypted rounds. Therefore, the dynamic rounds chaotic block cipher can guarantee the security of information transmission and realize the lightweight of the cryptographic algorithm.

## 1. Introduction

Cryptography is the core of information security technology, which is mainly used to ensure the confidentiality and integrity of information in the transmission process [1]. As an important branch of cryptography, block cipher is characterized by its quick speed, good scalability, strong adaptability, easy standardization, and convenient for hardware and software implementation, which not only has the application in the network security, message authentication, digital signature, etc., but also is considered as an ideal choice for information encryption in the “Internet+” domain [2]. In general, the design criteria of the block cipher can be summarized as the principle of confusion and diffusion, the simple principle of algorithm implementation, the principle of similarity and validity of encryption/decryption, and the principle of flexibility and transparency of encryption algorithm.

The encrypted rounds of block cipher generally depend on the compromise option between cryptographic security and operational efficiency. In most cases, the security of the block cipher will be enhanced by the increase of encrypted rounds. However, if there are too many encrypted rounds, the practicality of the block cipher will be affected. The Feistel structure, which is a classical and the most used block cipher structure, has the advantages of easy implementation, rapid diffusion, simple calculation and good security, etc. At present, the same encrypted rounds are used for all plaintext information by the Feistel structure block cipher, and it can easily lead to information leakage and decipherment due to the insufficient security of encryption of important information, and unnecessary computational burden and resource consumption caused by the excessive security of encryption of non-important information. In response to the above problems, a dynamic rounds chaotic block cipher based on keyword abstract extraction is proposed in this paper.

On the basis of traditional Feistel structure chaotic block cipher, the abstract extraction function in the Natural Language Processing and Information Retrieval Sharing Platform (NLPIR) is used to mark important information of text plaintext automatically before block encryption. Under condition of the same cryptographic structure and components, for a small number of plaintext blocks containing important information, the encrypted rounds of which would dynamically change within the interval [16,30], while for a large number of plaintext blocks containing non-important information, the encrypted rounds of which would dynamically change within the interval [8,15]. In each block encryption, the specific number of encrypted rounds is determined by the initial master key and the chaotic *S*-box. The randomness of the chaotic sequences is employed for DNA encoding, operation, and decoding to generate and extend keys. The fireworks algorithm is used to search for the optimal chaotic sequence explosively to construct the *S*-box. The dynamic change of the number of encrypted rounds not only enhances the complexity of the cipher but also reduces the total number of the encrypted rounds. By adopting such a block cipher algorithm that is dynamically encrypted according to different information, it is possible to guarantee the security of information transmission and realize the lightweight of the cryptographic algorithm.

The main contents of this paper are arranged as follows: the first part explains the background and significance of the research. The second part analyzes the research basis and status of the chaotic block cipher. The third part introduces the automatic abstract extraction based on keywords in the NLPIR big data search and mining development platform. The fourth part presents the system design of the dynamic rounds chaotic block cipher based on keyword abstract extraction, which includes the cryptographic structure, cryptographic components and the realization method of dynamic encrypted rounds. In the fifth part, the system performances of the dynamic rounds chaotic block cipher are analyzed and tested, and the security and practicality are evaluated according to the experimental data. The contents of this paper are summarized and concluded in the sixth part.

## 2. The Chaotic Block Cipher

The chaotic system has some typical characteristics, such as sensitive dependence of initial parameters, topological transitivity, tensile folding transformation characteristics, mixing, periodic point, ergodicity, positive Lyapunov exponent, fractional dimension, etc. [3]. With the further study of the chaotic theory, it is found that there are a natural relation and certain structure similarity between chaos and block cipher. Firstly, the iterative operation of the chaotic system is similar to the round function of the block cipher. Secondly, the block cipher can realize confusion and diffusion by the round encryption, and the chaotic system can spread the initial domain to the whole phase space through iteration. Thirdly, the encryption sensitivity of the block cipher is determined by the key, and the dynamical characteristics of the chaotic system are determined by the initial value and the system parameter [4,5].

At present, the main factors hindering the development of chaotic block cipher are as follows. Firstly, the chaotic theory is in the initial stage and the content of which is not sufficient. Secondly, the structural design of the chaotic block cipher is still in the exploratory stage. Thirdly, the finite operation precision effect results in the degradation of the dynamic characteristics of the chaotic system [6,7]. In addition, according to our previous research in the literature [8], it can be concluded that the dynamic characteristics of some chaotic systems are not ideal, which would lead to a weak key appearing in the cryptographic system if the parameter selection is not proper, and the algorithm would be invalid once the system track falls into the fixed point. Therefore, it is important to investigate how to find the chaotic system with excellent performance and better use of chaotic characteristics to design a block cipher.

Based on the analysis of chaotic systems that have been proposed so far, we have roughly divided the chaotic systems into two categories in the literature [9]. Discrete chaos is a system described by the difference equation, which can produce a chaotic signal of discrete time and continuous amplitude, such as Logistic, Tent and other low-dimensional chaotic maps. Continuous chaos is a system described by the differential equation, which can produce a chaotic signal of continuous time and amplitude, for example, Lorenz, Chen and other high-dimensional chaotic systems. Although the low-dimensional discrete chaotic maps have been widely researched and applied due to their simple mathematical model and easy hardware implementation, the disadvantages of which are apparent, such as small surjective map interval, narrow parameter ranges, and low complexity, etc. [10]. In view of the above problems, the two-stage cascaded chaotic map and three-dimensional Lorenz chaotic system are used to realize the key expansion and *S*-box generation in this paper.

Aiming at the deficiencies of the one-dimensional discrete chaotic map, we have proposed a two-stage cascaded chaotic map in the literature [11], which is constructed by cascading one-dimensional discrete chaotic maps Logistic and Tent. Owing to each iterative result of the cascaded chaotic map is determined by two chaotic maps together, which makes the iterative way more complex. Through comparing with a one-dimensional discrete chaotic map, it can be concluded that the two-stage cascaded chaotic map not only extends the surjective map interval but also has more complex dynamic characteristics and better pseudorandom performance, thus providing a reliable guarantee for the security of keys and cipher.

The difference iterative equation of the cascaded chaotic map is given to be

(1) $$x_{n+1}=1-\left|1-\mu_{1}\mu_{2}x_{n}\left(1-x_{n}\right)\right|.$$

In Equation (1), μ is the system parameter, $x_{n}$ is the initial value. When μ∈(1,2), $x_{n}\in \left(0,1\right)$, the cascaded chaotic map is in the chaotic state.

Lorenz is a classical three-dimensional continuous chaotic system. Apart from being non-periodic, unconvergent but bounded, and extremely sensitive to initial values, it also has the following three advantages: complex system structure makes it more difficult to predict output chaotic sequence, the solution space constituted of three initial values and three parameters is significantly higher than the low-dimensional discrete chaotic maps, and the application of three chaotic sequences makes the design of *S*-box more flexible [12,13].

The three-dimensional first order nonlinear differential equation of the Lorenz chaotic system is as follows:

(2) $$\begin{array}{c} \frac{dx}{dt}=a\left(y-x\right),\\ \frac{dy}{dt}=\left(bx-y-xz\right),\\ \frac{dz}{dt}=\left(xy-cz\right). \end{array}$$

In Equation (2), *a*, *b* and *c* are the system parameters. When *a* = 10, *b* = 28, *c* = 8/3, the Lorenz chaotic system is in the chaotic state [14].

## 3. The Keyword Abstract Extraction

NLPIR is a set of software designed for processing the original text, which combines natural language understanding, web search, text mining technologies, and provides 11 functions, such as word segmentation tagging, abstract and keyword automatic extraction, big data clustering and hotspot analysis, big data classification filtering, and HyperText Markup Language (HTML) text extraction [15]. In this paper, the entity annotation of important information in the plaintext of text is realized through the automatic abstract extraction based on keywords.

Automatic abstract extraction based on keywords is shown in Figure 1. The input text is converted into canonical text by preprocessing, and the double-array Trie tree is used to filter the stop words and construct word segmentation dictionary, so as to obtain the sentence sequence of the text and the word sequence expression of the sentence. By calculating the weights of words and sentences, all the sentences in the text are arranged in the order of decreasing weight; the degree of redundancy of abstract is reduced by effectively remove duplication that has inclusion relation in the text. Finally, the sentences with the highest weights are identified as abstract sentences, which are marked in the order in which they appear in the text.

### 3.1. The Keyword Extraction

The basis of the keyword extraction is the weight of a word, which is mainly analyzed by the factors of the word frequency-inverse text frequency, the adjacent category value, the positional locality, and the position of the word in the sentence, etc. [16].

1. The word frequency-inverse text frequency is used to calculate the weight of a word. Creating a plaintext corpus in text format, the word frequency reflects the word feature by counting the frequency of each word appearing in a text. The inverse text frequency reflects the importance of a word by counting the frequency of which appearing in all texts of the corpus, then the word feature expressed only by word frequency would be corrected. The ratio of the frequency of a feature word appearing in the text to the number of texts containing that feature word in the corpus is taken as the weight of the word, i.e.,
  (3) $$\omega \left(t,d\right)=\log\left(tf\left(t,d\right)+1.0\right)\times \log\left(N/n_{t}+1.0\right).$$
  In Equation (3), ω(t,d) is the weight of a feature word *t* in the text *d*, tf(t,d) is the frequency of a feature word *t* appearing in the text *d*, *N* is the number of all texts in the corpus, and $n_{t}$ is the number of texts in which *t* appears in the corpus. The more frequently a word appears in a text and the less frequently it appears in other texts simultaneously, which indicates that the word has more distinguishable ability for expressing this text, so the weight value should be greater.
2. The keywords with higher word frequency are extracted using the adjacent category value of words. The left adjacent category value of a string is the number of words appearing on the left side of that, and the right adjacent category value of a string is the number of words appearing on the right side of that. The total adjacent category value of a string refers to the number of words appearing on the left and right sides of that. The greater the adjacency category value of a word, the more flexible it is to use, and the more likely it is to use in different contexts, the higher the probability to become a keyword.
3. The positional locality of words is used to extract keywords with lower word frequency. The positional variance of the word appearing in the text is used to represent the positional locality of that. Assuming that the candidate word *T* appears *n* times in the corpus, and the appearing positions of candidate word *T* are $P_{1},P_{2},\ldots ,P_{n}$ respectively; then, the positional variance D(*T*) of *T* is
  (4) $$D\left(T\right)=\frac{\sum_{i=1}^{n}{\left(P_{i}-\bar{P}\right)}^{2}}{n-1}.$$
  In Equation (4), $\bar{P}$ represents the average position of the candidate word *T*. The positional variance of the word indicates the sparsity of the distribution of word. The smaller the positional variance, the higher the degree of concentration in each position and the stronger the locality of the string. Assuming that LE represents a local measure value of a string, the locality formula is
  (5) LE(T)=1/D(T).
4. A piecewise function is used to quantify the influence of sentence position on the weight of the word, i.e.,
  (6) $$SP=\left\{\begin{array}{c} -\alpha (x-l/2),x<l/2,\\ \beta (x-l/2),x>l/2. \end{array}\right.$$
  In Equation (6), *x* represents the position of the sentence in the paragraph, *l* represents the number of sentences in the paragraph, α represents the smoothing factor of the sentence position in the upper half of the paragraph, and β represents the smoothing factor of the sentence position in the lower half of the paragraph.
5. The final weight of a word is obtained by a weighted sum based on the above features. The weighting coefficient of each feature is obtained through artificial training of a data set.

### 3.2. The Sentence Weight and Similarity Calculation

The key to the entity annotation of important information is the selection of sentences, the principle of which is to use the most important sentence to reflect the topic of the text as much as possible, that is, the relevance of the topic is as high as possible. Sentence selection is divided into sentence weight calculation [17] and sentence similarity calculation [18]. The commonly used calculation models of sentence similarity include mainly the vector space, the query likelihood, and the translation models, where the vector space model is widely used due to its simple calculation.

In the vector space model, sentences are taken as vectors, and each dimension of the vector represents a segment of the sentence. The granularity of the segment can be a single word or phrase, *N*-tuple, etc., which are collectively called the lexical item and denoted by *t*; each element of the vector represents the weight of the lexical item, which is expressed by ω. The sentence S can be represented as a vector $\left(t_{1},\omega_{1};t_{2},\omega_{2};\ldots ;t_{N},\omega_{N}\right)$, the lexical item *t* is called the feature, and the weight of which as the feature weight, which is abbreviated as $S(\omega_{1},\omega_{2},\ldots ,\omega_{N})$. Sentences are expressed as the vector space model, and features and feature weights need to be determined.

The similarity is defined as the cosine of the angle between two vectors

(7) $$sim\left(Q,D\right)=\frac{\sum_{t\in Q\cap D}\omega_{d,t}\omega_{q,t}}{\sqrt{\sum_{t\in Q}\omega_{q,t}^{2}\sum_{t\in D}\omega_{d,t}^{2}}}.$$

### 3.3. The Sentence Redundancy Calculation

In order to reduce the redundancy of the abstract content as much as possible, the cosine similarity is used to express the sentence redundancy between the candidate abstract sentence and the abstract sentence. A candidate abstract sentence $s_{i}$ and abstract sentence set *S* consists of two parts:

One is the maximum redundancy between the candidate abstract sentence and the sentence in abstract sentence set,

(8) $$R_{\max}=\max_{s_{j}\in s}sim\left(s_{i},s_{j}\right).$$

The other is the average redundancy between the candidate abstract sentence and all the sentences in abstract sentence set,

(9) $$\bar{R}=\frac{\sum_{m}^{j=1}sim(s_{i},s_{j})}{m}.$$

In Equation (9), *m* is the total number of sentences in *S*.

The weight of the candidate abstract sentence is recalculated as

(10) $$W^{\prime }=\lambda W-\left(1-\lambda \right)\left(\alpha R_{\max}+\left(1-\alpha \right)\bar{R}\right).$$

In Equation (10), *W* is the sentence weight calculated only according to the weights of words in the sentence, which will not change when the sentence redundancy is calculated multiple times. λ and α are adjustment coefficients, which are generally obtained through training by a certain training set.

All candidate abstract sentences are reordered according to their weights; the candidate sentence with the largest weight is found and marked as an abstract sentence if it meets the requirement of the length of the abstract. In the same way, the other candidate abstract sentences are judged in turn.

## 4. The Dynamic Rounds Chaotic Block Cipher

### 4.1. The Cryptographic Structure

**Figure 2:**
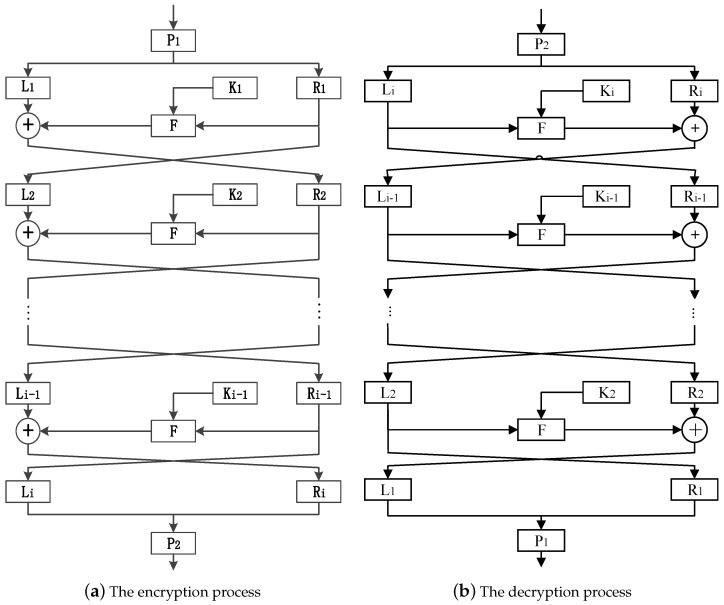
The dynamic rounds chaotic block cipher of Feistel structure.

The dynamic rounds chaotic block cipher of the Feistel structure is shown in Figure 2. One block of plaintexts is averagely divided into two groups after scrambled by $P_{1}$-box, which can be denoted as $L_{i}$ and $R_{i}$, respectively. Under the action of the round key $K_{i-1}$, $R_{i-1}$ passes through the *F* function and does the exclusive OR (XOR) operation with $L_{i-1}$ to generate new $R_{i}$, while $R_{i-1}$ becomes new $L_{i}$. Finally, the ciphertexts are outputted through the scrambling of -box after round-by-round iteration. The above encryption process can be expressed as

(11) $$\begin{array}{c} L_{i}=R_{i-1},\\ R_{i}=L_{i-1}⊕F\left(R_{i-1},K_{i-1}\right). \end{array}$$

In Equation (11), *F* is the round function, *i* is the number of encrypted rounds, $K_{i-1}$ is the round key, and ⊕ is XOR operation.

The encryption and decryption process of dynamic rounds chaotic block cipher of Feistel structure is similar, and decryption is the inverse operation of encryption. It does not need to do the inversion because the round functions of encryption and decryption are the same, which makes the design of round function more flexible. The cryptographic structure is relatively simple and easy to implement.

### 4.2. The Round Function

**Figure 3:**
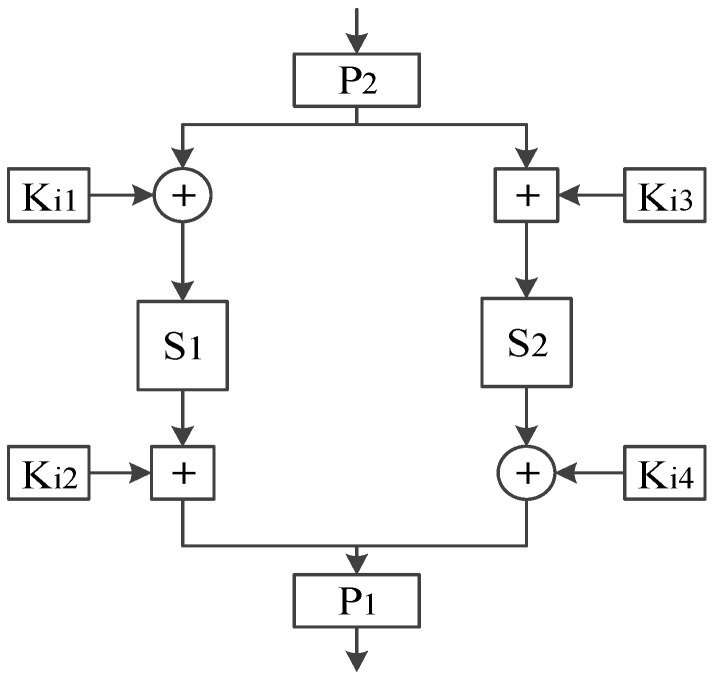
The round function *F* of the dynamic rounds chaotic block cipher.

As shown in Figure 3, the round function *F* is the core to realize the confusion and diffusion of the block cipher, which is composed of the round key $K_{i1}-K_{i4}$, the double *S*-boxes $S_{1}$ and $S_{2}$, the double *P*-boxes $P_{1}$ and $P_{2}$, XOR and modular addition operations. In each round of information encryption, the input $R_{i}$ is replaced in reverse order by $P_{2}$-box, the output of which is divided into left and right groups on averagel, and do the XOR and modular addition operations with round keys of $K_{i1}$ and $K_{i3}$ respectively. Then, the information confusion is achieved through two different $8\times 8S_{1}$-box and $S_{2}$-box, the output of which do the modular addition and XOR operations with round keys of $K_{i2}$ and $K_{i4}$, respectively. After combination, the output of the round function *F* is obtained through the right shift operation of $P_{1}$-box finally.

As the only nonlinear component, the *S*-box is the key to design round function *F*. In the present design of *S*-box based on Lorenz chaotic system, the method of Runge–Kutta has been used to solve the Lorenz chaotic system and then select a chaotic sequence randomly to construct the *S*-box, which makes it difficult to guarantee the rationality and superiority of the selected chaotic sequence. The fireworks algorithm [19] has high optimization precision and convergence speed through inheriting and approximating the local information of the neighborhood to search solution space explosively. Therefore, it is used to search the optimal chaotic sequence from the *x*-axis, *y*-axis outputs of Lorenz chaotic system, and combines with reverse coding operation and edge contraction matrix transformation to construct $S_{1}$-box and $S_{2}$-box, so as to provide a reliable guarantee for the cryptographic performance of the round function *F*.

### 4.3. The Key Expansion

The master key $K_{i}$ is an essential part in each round of information encryption. The cascaded chaotic sequence generated by Equation (1) is digitally quantized and represented as $T_{i}$, which would be, on average, divided into three groups to do the DNA decision and encoding. As shown in Table 1, a DNA sequence consists of four different nucleotide bases: adenine (A), thymine (T), cytosine (C) and guanine (G) [20]. Using the randomness of the digital cascaded chaotic sequence, the type of DNA encoding is determined according to the value of the first three bits of each sequence. As shown in Table 2, after addition and extension operations are performed on the three groups of DNA sequences, the 32-bit base sequence is decoded by DNA and the initial master key $K_{i}$ can be output.

In the iterative operation of the round function, each round of the master key $K_{i}$ needs to generate four round subkeys through a certain key expansion algorithm, which is a vital factor affecting the encryption performance of block cipher. In this paper, the initial master key $K_{i}$ is, on average, divided into four groups to do the DNA decision and encoding. On the basis of the base complementary transformation, which is A and T complementary pairing, C and G complementary pairing, and then the round subkeys will be output after DNA decoding. By following the above rules, the subkeys $K_{i1}K_{i2}K_{i3}K_{i4}$ required for each round of encryption can be generated one by one.

### 4.4. The Dynamic Rounds

Considering the security, speed, storage space and other factors, a kind of dynamic generation of encrypted rounds within two intervals of [16,30] and [8,15] is designed by using the randomness of chaotic sequence and the nonlinearity of chaotic *S*-box, which will achieve different levels of dynamic encryption for important and non-important plaintext blocks respectively. The 4 × 4 *S*-box generated by the Lorenz chaotic system and fireworks algorithm is shown in Table 3. When the input data is four bits, the first two bits determine the row of *S*-box, and the rear two bits determine the column of *S*-box, so any four-bit binary input can be converted to any decimal number in the interval of [0,15].

In the encrypted process of each block, it has to calculate the number of dynamic rounds needed to encrypt firstly. The first four bits of the initial master key are used as the input to the *S*-box, and an output of the *S*-box is set to $l_{i}$. Then, the dynamic rounds of $L_{i}$ and $L_{j}$ in the intervals of [8,15] and [16,30] are respectively expressed as

(12) $$L_{i}=\left\{\begin{array}{cc} l_{i}+8, & l_{i}<8,\\ l_{i}, & l_{i}\geq 8, \end{array}\right.$$

(13) $$L_{j}=\left\{\begin{array}{cc} 2(l_{j}+8), & l_{j}<8,\\ 2l_{j}, & l_{j}\geq 8. \end{array}\right.$$

As a kind of symmetric cipher, block cipher encryption and decryption use the same key, the number of rounds that the block has been encrypted can be determined according to the same way in the decryption, so that the plaintext information can be recovered.

## 5. The Analysis and Test of Dynamic Rounds Chaotic Block Cipher

Linear analysis [21] and differential analysis [22] have always been the most effective attacking means for the block cipher, and are also important indicators for block cipher design and security assessment. When evaluating the linear and differential safety features of the Feistel structure, the literature [23] pointed out that, if the upper bound of the maximum linear and differential characteristic probability is less than a safety threshold, it shows that these two analyses are actually safe. The literature [21,22] discusses in detail the practical methods for evaluating the ability of anti-linear and differential analysis of cipher. That is, the upper bounds of the maximum linear and differential approximation probabilities are calculated by the number of active *S*-boxes, whose method is used when evaluating linear and differential cryptanalysis of block ciphers such as AES, Camellia, and CLEFIA.

### 5.1. The Linear Analysis

The linear analysis is a known-plaintext attack, whose basic principle is to seek an effective linear approximation between plaintext, ciphertext and key. When the linear deviation of the approximation is large enough, the partial key information can be inferred from a certain amount of plaintext and ciphertext pairs.

For any given $a\in F_{2}^{n}$, $b\in F_{2}^{n}$, it is defined that the linear approximation probability of *S*-box whose input and output are *n* bits,

(14) $$LP^{S}\left(a\to b\right)={\left(\frac{\#\left\{x\in F_{2}^{n}\left|x\cdot a=S(x)\cdot b\right.\right\}-2^{n-1}}{2^{n-1}}\right)}^{2}.$$

The maximum linear approximation probability [24] of the *S*-box is defined as

(15) $$LP_{\max}^{S}=\max_{a,b\neq 0}{\left(\frac{\#\left\{x\in F_{2}^{n}\left|x\cdot a=S(x)\cdot b\right.\right\}-2^{n-1}}{2^{n-1}}\right)}^{2}\leq \frac{L_{S}\left(a,b\right)}{2^{n}}.$$

In Equation (15), $F_{2}^{n}$ represents the *n*-dimensional vector space on the binary domain, that is, the set of all possible inputs of *x*, $2^{n}$ is the number of all elements in the set $F_{2}^{n}$, and a·b represents the inner product operation between two operands of *a*, *b*. $L_{S}\left(a,b\right)$ is the degree of linearity of the *S*-box, and its relationship with the degree of nonlinearity $L_{S}\left(a,b\right)=2^{n-1}-NL_{S}\left(a,b\right)$ is given in literature [25]. $LP_{\max}^{S}$ can be regarded as the maximum probability that any given b≠0 traverses all the values *x* to get the output as *a*. Therefore, the smaller the value of the maximum linear approximation probability of the *S*-box, the stronger the ability to resist the linear attack.

The linear active *S*-box [26] refers to an *S*-box whose output representation vector is non-zero. Then, the number of minimum linear active *S*-boxes is

(16) $$n_{l}=\min_{\Gamma z^{\prime }\neq 0}\left\{w_{H}\left(\Gamma z\right)+w_{H}\left(\Gamma z^{\prime }\right)\right\}.$$

In Equation (16), $\Gamma z^{\prime },\Gamma z$ are the input/output representation vectors of the linear diffusion layer, $w_{H}$ represents the Hamming weight, and it can be calculated that the number of linear active *S*-boxes when encrypting eight rounds is 56.

Since the average degree of nonlinearity of the *S*-box in this paper is 106, the maximum linear approximation probability of the *S*-box is calculated to be that $LP_{\max}^{s}=2^{-3.54}$, so it can be obtained the maximum linear approximation probability of the eighth round is as follows:

(17) $$MLP_{\max}^{8L}\leq {\left(LP_{\max}^{S}\right)}^{LS}=2^{-198}<2^{-64}.$$

In Equation (17), *L* represents the number of encrypted rounds and LS represents the number of linear active *S*-boxes. When the block length of the block cipher is 64 bits, according to the linear complexity analysis, if the number of encrypted rounds is greater than 8, the maximum linear approximation probability would be less than the upper bound of $2^{-64}$, it would be difficult to find an effective linear feature for analysis. Therefore, it can be considered that the dynamic round interval in this paper is not only practically safe for linear analysis but also has a certain safety margin.

### 5.2. The Differential Analysis

The differential analysis is a kind of chosen-plaintext attack. Its basic idea is to recover some or all of the keys by analyzing the impact of the differences of plaintext pairs on the difference of ciphertext pairs.

For any given Δx and Δy, it is defined that the differential approximation probability of *S*-box whose input and output are *n* bits,

(18) $$DP^{S}\left(\Delta x\to \Delta y\right)=\frac{\#\left\{x\in F_{2}^{n}\left|S(x)⊕S(x⊕\Delta x)=\Delta y\right.\right\}}{2^{n}}.$$

The maximum differential approximation probability [27] of the *S*-box is defined as

(19) $$DP_{\max}^{S}=\max_{\Delta x\neq 0,\Delta y}\left(\frac{\#\left\{x\in F_{2}^{n}\left|S(x)⊕S(x⊕\Delta x)=\Delta y\right.\right\}}{2^{n}}\right)\leq \frac{Diff(S)}{2^{n}}.$$

In Equation (19), $Diff\left(S\right)$ is the difference uniformity of the *S*-box, $DP_{\max}^{S}$ can be regarded as the maximum probability that any given Δx≠0 traverses all the values *x* to get the output as Δy. Therefore, the smaller the value of the maximum differential approximation probability of the *S*-box, the stronger the ability to resist the differential attack [24].

The differential active S box [26] refers to an *S*-box whose input difference is non-zero. Then, the number of minimum differential active *S*-boxes is

(20) $$n_{d}=\min_{\Delta z\neq 0}\left\{w_{H}\right.\left(\Delta z\right)+\left.w_{H}\left(\Delta z^{\prime }\right)\right\}.$$

In Equation (20), $\Delta z,\Delta z^{\prime }$ are differential input and output of the linear diffusion layer, $w_{H}$ represents Hamming weight, and it can be calculated that the number of differential active *S*-boxes when encrypting eight rounds is 42.

Since the maximum value of the differential uniformity of *S*-box in this paper is 10, the maximum differential approximation probability of the *S*-box is calculated to be that $DP_{\max}^{s}=2^{-4.68}$, so the maximum differential approximation probability of the eighth round can be obtained as follows:

(21) $$MDP_{\max}^{8L}\leq {\left(DP_{\max}^{S}\right)}^{DS}=2^{-196}<2^{-64}.$$

In Equation (21), *L* represents the number of encrypted rounds, and DS represents the number of differential active *S*-boxes. From this, it can be seen that there is no valid difference feature for analysis if the number of encrypted rounds is greater than 8, and the dynamic rounds chaotic block cipher proposed in this paper can resist the differential attack.

The randomness of ciphertext is an important index to measure the performance of cryptographic algorithms. If the distribution of ciphertext is not uniform or random enough, the cryptanalyst can break and obtain information through the ciphertext-only attack. In order to reflect the encryption performance of dynamic rounds chaotic block cipher as accurately as possible, the information entropy, “0–1” balance and NIST tests of ciphertext are carried out respectively in this paper.

### 5.3. The Information Entropy Test

The probability of information appearing in the ciphertext is called information entropy, which is used to indicate the confusion degree of the ciphertext, and its calculation equation is

(22) $$H\left(s_{i}\right)=\sum_{i=0}^{2^{n}-1}p\left(s_{i}\right)\log_{2}\frac{1}{p(s_{i})}.$$

In Equation (22), the probability that a character $s_{i}$ appears is denoted as $p(s_{i})$. If the plaintext contains $2^{n}$ characters, the ideal value of its information entropy should be *n*.

For the test results of information entropy of different block ciphers as shown in Table 4, the test performance of information entropy of dynamic rounds chaotic block cipher is optimal compared with other traditional block ciphers, the ciphertext information of which can be more sufficiently confused and diffused.

### 5.4. The “0–1” Balance Test

The “0–1” balance refers to the relationship between the number of 0 and 1 in the ciphertext, which is used to indicate the randomness degree of ciphertext, and its calculation equation is

(23) $$\varepsilon =\frac{|k_{1}-k_{2}|}{n}.$$

In Equation (23), $k_{1}$ and $k_{2}$ represent the number of 0 and 1, respectively. *n* is the total number of 0 and 1. The more ε gets closer to 0, the better the “0–1” balance and the stronger the randomness of ciphertext.

For the test results of “0–1” balance of different block ciphers as shown in Table 5, the “0–1” balance is closer to 0 as the ciphertext length increases, and the test performance of “0–1” balance of dynamic rounds chaotic block cipher is obviously better than other traditional block ciphers, which shows that it can hide the plaintext information more thoroughly and has stronger anti-analysis ability.

### 5.5. The NIST Test

In order to further verify the statistical characteristics of ciphertext, the SP800-22 test package developed by the National Institute of Standards and Technology (NIST) is used for the random performance detection [28]. In this paper, single-bit frequency test, block frequency test, run test and block long run test are chosen for the random test. The greater the *p*-value, the better the randomness of the test sequences.

As shown in Table 6, the ciphertexts of the dynamic rounds and other traditional block ciphers can all pass the NIST test. In comparison, the test performance of dynamic rounds chaotic block cipher is optimal, which shows that the dynamic change of encrypted rounds can not only reduce the amount of calculation but also better resist ciphertext-only attack.

### 5.6. The Performance Analysis

**Figure 4:**
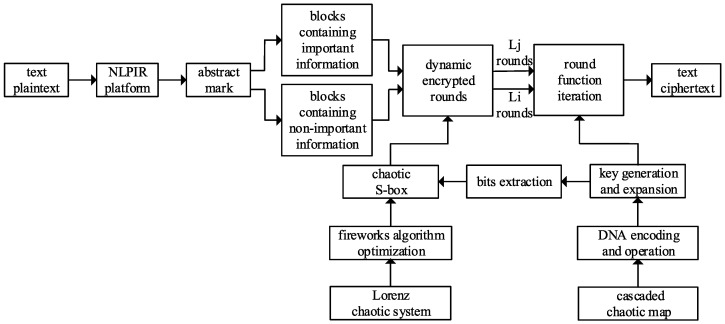
The description of the proposed encryption cipher.

**Figure 5:**
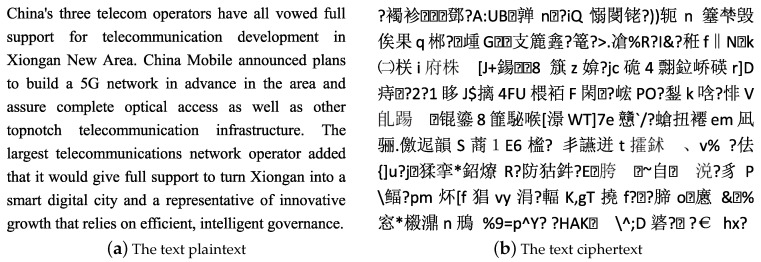
The example of the proposed encryption cipher.

The description of the proposed encryption cipher is shown in Figure 4. The randomness of the chaotic sequence is employed for DNA encoding, decoding, and operations, random groups similar to DNA sequences are constructed to generate and extend keys. The fireworks algorithm is adopted to explosively search the optimal chaotic sequence from the outputs of the Lorenz chaotic system and to construct *S*-box through inheriting and approximating the local information of the neighborhood. the randomness of chaotic sequence and the nonlinearity of chaotic *S*-box are used for the dynamic generation of encrypted rounds in two intervals, realizing more numbers of dynamic rounds encryption for the important information blocks marked by NLPIR, while fewer numbers of dynamic rounds encryption for the non-important information blocks that are not marked. The example of the proposed encryption cipher is shown in Figure 5.

The ideal lightweight cipher should occupy a small circuit area, consume the least amount of resources and power, and perform as fast as possible. The complexity of implementation is one of the most dominant factors that affect the design approach of the cipher. It is determined by the logic gates that are required to implement a cipher, and the relevant metric is called Gate Equivalent (GE). Software implementations are optimized for throughput and energy consumption by requiring fewer cycles since voltage and clock frequency are usually fixed for microcontrollers. The performances of most lightweight block ciphers are evaluated in the literature [29]. Table 7 shows the results of comparison between this algorithm and several of them, which indicates that it meets the indexes of lightweight block ciphers in terms of occupied area and throughput and can be used in environments with limited resources.

## 6. Conclusions

Considering the trade-off between security and performance, in the case of the same encrypted structure, process and round function, the dynamic rounds chaotic block cipher proposed in this paper based on the automatic abstract extraction function in the NLPIR big data search and mining development platform, which will achieve different levels of dynamic encryption for important and non-important plaintext information, respectively. Compared with traditional and lightweight block ciphers, the dynamic variety of encrypted rounds can realize the security of information transmission and the lightweight of the cryptographic algorithm. At the same time, the research and application of the dynamic rounds chaotic block cipher have important significance and research value to the design analysis theory of traditional block cipher and the further development of chaotic application theory.

## Figures and Tables

**Figure 1:**
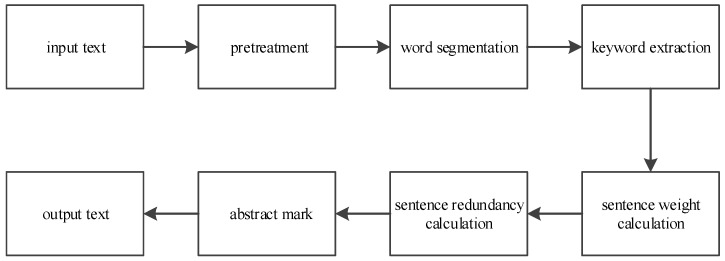
The automatic abstract extraction based on keywords.

**Table 1:** DNA decision and encoding rules.

Digital Chaotic Sequence	DNA Encoding Type	A	T	C	G
000	1	00	10	01	11
001	2	00	01	10	11
010	3	01	11	00	10
011	4	01	00	11	10
100	5	10	11	00	01
101	6	10	00	11	01
110	7	11	10	01	00
111	8	11	01	10	00

**Table 2:** DNA addition operation.

+	A	T	C	G
A	C	G	A	T
T	G	C	T	A
C	A	T	C	G
G	T	A	G	C

**Table 3:** 4 × 4 *S*-box.

	0	1	2	3
0	13	14	8	6
1	1	12	11	7
2	2	0	4	5
3	10	9	3	15

**Table 4:** The test results of information entropy.

Information Entropy	5000 Bit	10,000 Bit	100,000 Bit	240,000 Bit
dynamic rounds	7.99997468	7.99999863	7.99999965	7.99999995
RC5	7.96834100	7.99034700	7.99994000	7.99990800
RC6	7.97544600	7.99237500	7.99912200	7.99992600
AES	7.97581500	7.99258200	7.99928300	7.99992300
Skipjack	7.96970000	7.98759000	7.99923100	7.99993500
WSN_DMC	7.97496900	7.99376100	7.99904500	7.99989600
WSN_CSB	7.97503800	7.99088700	7.99895900	7.99991800

**Table 5:** The test results of “0–1” balance.

“0–1” Balance	5000 Bit	10,000 Bit	100,000 Bit	240,000 Bit
dynamic rounds	0.001600	0.000800	0.000079	0.000033
RC5	0.024850	0.021800	0.048040	0.004172
RC6	0.016000	0.020200	0.056280	0.004506
AES	0.025200	0.035240	0.031500	0.001782
Skipjack	0.016200	0.151380	0.022840	0.001948
WSN_DMC	0.021200	0.005600	0.000220	0.000376
WSN_CSB	0.009680	0.021400	0.002560	0.001080

**Table 6:** The test results of NIST.

Test Items	Single-Bit Frequency Test		Block Frequency Test		Run Test		Block Long Run Test	
	Proportion	*p*-Value	Proportion	*p*-Value	Proportion	*p*-Value	Proportion	*p*-Value
dynamic rounds	100/100	0.866429	99/100	0.68492	100/100	0.98539	99/100	0.869738
MCS ^1^	98/100	0.494392	99/100	0.595549	99/100	0.304126	100/100	0.437274
LCS ^2^	99/100	0.304126	99/100	0.911413	98/100	0.657933	99/100	0.262249

^1^ mixed chaotic block cipher scheme (MCS); ^2^ Logistic chaotic block cipher scheme (LCS).

**Table 7:** The performance comparison with other block ciphers.

Cipher	Block Size (Bits)	Key Size (Bits)	Tech (µm)	Area (GE)	Latency (Cycles/Block)	Throughput at 100 KHz (Kbps)
dynamic rounds	64	64	0.18	1957	19	336
HIGHT	64	128	0.25	3048	34	188
PRESENT-80	64	80	0.18	1570	32	200
Piccolo	64	80	0.13	1136	27	237
LBlock	64	80	0.18	1320	32	200
Camellia	128	80	0.18	6511	44	290
TWINE	64	128	0.09	1866	36	178
LBLOCK	64	80	0.18	1320	32	200
MIBS	64	80	0.18	1530	32	200
